# Thermal Infrared Pedestrian Image Segmentation Using Level Set Method

**DOI:** 10.3390/s17081811

**Published:** 2017-08-06

**Authors:** Yulong Qiao, Ziwei Wei, Yan Zhao

**Affiliations:** 1 School of Information and Communication Engineering, Harbin Engineering University, Harbin 150001, China; qiaoyulong@hrbeu.edu.cn; 2 School of Mechatronics Engineering, Harbin Institute of Technology, Harbin 150001, China; 16s008206@stu.hit.edu.cn

**Keywords:** thermal pedestrian images, active contour model, level set method, one-bit transform, edge indicator function

## Abstract

The edge-based active contour model has been one of the most influential models in image segmentation, in which the level set method is usually used to minimize the active contour energy function and then find the desired contour. However, for infrared thermal pedestrian images, the traditional level set-based method that utilizes the gradient information as edge indicator function fails to provide the satisfactory boundary of the target. That is due to the poorly defined boundaries and the intensity inhomogeneity. Therefore, we propose a novel level set-based thermal infrared image segmentation method that is able to deal with the above problems. Specifically, we firstly explore the one-bit transform convolution kernel and define a soft mark, from which the target boundary is enhanced. Then we propose a weight function to adaptively adjust the intensity of the infrared image so as to reduce the intensity inhomogeneity. In the level set formulation, those processes can adaptively adjust the edge indicator function, from which the evolving curve will stop at the target boundary. We conduct the experiments on benchmark infrared pedestrian images and compare our introduced method with the state-of-the-art approaches to demonstrate the excellent performance of the proposed method.

## 1. Introduction

Infrared imaging has been applied in many application fields, such as industrial inspection, defense and security. Therefore, infrared target detection, recognition and tracking are important topics in infrared image processing, in which the infrared image segmentation is one of the fundamental steps. In the computer vision and image processing fields, various methods have been proposed to solve the image segmentation problems [1,2,3]. However, due to the particular properties of infrared images, infrared image segmentation is still a challenging problem.

Active contour models have been applied in image segmentation in recent decades because they are able to provide smooth and closed boundary contours as segmentation results. The level set method (LSM) for capturing moving fronts was proposed by Osher and Sethian [4]. In computer vision and image processing, the level set method was introduced independently by Caselles et al. [5,6,7] and Malladi et al. [8] in the context of the active contour (or snake) models [9,10] for image segmentation. In level set-based image segmentation methods, the boundary (contour or interface) of the region is represented as the zero level set of a level set function, and thus the moving contour is formulated as the zero level set of the evolving level set function. The level set method has several advantages, such as being able to represent interfaces with complex topology and dealing with the topology changes in the natural way. These advantages all help the level set method to be intensively explored in the image segmentation field.

In generally, level sets-based image segmentation methods can be classed into two categories, namely, region-based methods and edge-based methods. The region-based methods make use of some region descriptor to control the evolution of the active contour. Based on the model proposed by Mumford and Shah [11] and the assumption of intensity homogeneity, Chan and Vese introduced the two phase level set framework [12] and the multiphase level set framework [13] for image segmentation (also called piecewise constant model). Li et al. [14] proposed a region-based image segmentation method that is able to deal with the intensity inhomogeneity in the image segmentation via a bias field. Zhou et al. [15] represented a region fitting method to improve the Chan and Vese model for infrared image segmentation.

The edge-based methods utilize the edge information [16] for image segmentation, which shows potentially improved performance in various applications, for example, object extraction in aerial imagery [17,18], medical image segmentation [19,20,21,22,23], and infrared image segmentation [24,25]. Li et al. [26,27] and Zhang et al. [28] proposed the level set evolution without re-initialization. Both of them showed the effectiveness of their proposals in the medical image segmentation field. Meng Li et al. [29] introduced the tensor diffusion level set method to extract infrared target contours from a complex background, in which the structure tensor and eigenvalues are used to represent the edges of the infrared object. Tan et al. [24] took advantage of the background subtraction and the level set- based active contour model for human segmentation in infrared image sequences. Zhao et al. [30] proposed an edge map based on the guide filter and the gradient vector flow (GVF) to segment infrared images.

However, it is well known that the edge-based methods suffer from serious boundary leakage problems in images with weak object boundaries [31]. The reason may be that the edge-stop function cannot stop the contour evolution at the poorly defined (weak) boundaries. Therefore, Pratondo [19] proposed a method to construct the robust edge-stop function to improve the medical image segmentation performance. In infrared thermal imaging, the device collects the infrared radiation from the objects and the surrounding scene. In some cases, the temperature difference of some parts of the target and the surroundings is not remarkable, and (or) the differences of the infrared energy emitted from a target are significant, which may result in weak target boundaries and inhomogeneity intensity in the target region, respectively. The former may cause the boundary leakage problem, while the latter may lead to the level set evolution prematurely stopping, and then the boundary is formed in the interior of the target region. Pratonodo’s method [19] can be used to solve the boundary leakage problem, however, we have to select the training samples for training the classifier to construct the robust edge-stop function, which is difficult for object(s) with severely inhomogeneous intensities.

In order to deal with the boundary leakage problem and the intensity inhomogeneity, we propose herein a robust infrared image segmentation method, named intensity adjustment level set evolution (IALSE), which is based on using the level set evolution to extract infrared target contours. The introduced approach constructs a soft mark with a one-bit transform to enlarge the intensity changes around the edges, and then defines a weight function to adaptively adjust the image intensity so that the intensities in the interior of the target approach uniformity. Therefore, the edge-based contour evolution can stop at the desired boundary automatically.

The rest of this paper is organized as follows: in the second section, we give a necessary background of the edge-based level set method and our motivation. In the third section, we introduce our method to address the boundary leakage and the premature stop problems of the level set evolution, and then summarize the whole segmentation method. In Section 4, infrared image segmentation experiments are conducted to demonstrate the effectiveness of the proposed method. Finally, we conclude our work in Section 5.

## 2. Background and Motivation

### 2.1. Traditional Level Set Method for Image Segmentation

The level set was proposed for capturing moving fronts by Osher et al. [4]. Caselles et al. [5,6,7] proved that a particular of the classical energy of the snakes model is equivalent to finding a geodesic curve in a Riemannian space with a metric derived from the image content, and then introduced the geometric active contours, which is based on the idea that the contours are represented as the zero level set of an implicit function, called level set function.

In the context of image segmentation, let$\;C\left(t,q\right):\;\left[0,\infty \right)\times \left[0,1\right]\to R^{2}$ be a dynamic parametric contour and $I:\left[0,a\right]\times \left[0,b\right]\to R^{+}$ be a given image, in which t is a temporal variable and q is a spatial parameter. In order to deform the initial curve $C_{0}\left(q\right)=C\left(0,q\right)$ towards the object boundary, Caselles et al. [5] showed that it should follow the curve evolution equation:

(1) $$\frac{\partial C\left(t,q\right)}{\partial t}=FN$$

where N is the unit inward normal vector to the desired boundary (the local optimal curve), and F is the speed function that controls the motion of the curve along its normal direction. Assume that the curve C is a level set of a function $\phi ∶R^{+}\times R^{2}\to R$ such that:

(2) ϕ(t,x,y) =constant

This means that the embedding function ϕ is an implicit representation of the curve. Now we let ϕ be a signed distance function(|∇ϕ|=1), whose value at a point is equal to the signed value of the distance between that point and its closest point on the zero level set. Caselles et al. [5] demonstrated that if the curve C evolves according to Equation (1), then the embedding function ϕ should deform as follows:

(3) $$\frac{\partial \phi }{\partial t}=F\left|\nabla \phi \right|$$

The final level set representation of the geodesic active contours is:

(4) $$\frac{\partial \phi }{\partial t}=g\left(I\right)\left(c+k\right)\left|\nabla \phi \right|+\nabla \phi \cdot \nabla g\left(I\right)$$

in which c is a constant that can improve the convergence speed and allow the detection of non-convex objects. $k=\mathrm{div}\left(\frac{\nabla \phi }{\left|\nabla \phi \right|}\right)$ is the curvature. The stopping function g(I) is to stop the evolving curve when it arrives to the object boundary. Usually, g(I) is defined as follows:

(5) $$g\left(I\right)=\frac{1}{1+{\left|\nabla \hat{I}\right|}^{p}},\;p=1,2\ldots .$$

where $\hat{I}$ is the smoothed version of the image I. Generally, we utilize the Gaussian kernel $G_{\sigma }$ (σ is the standard deviation) to smooth the image as$\;\hat{I}=G_{\sigma }*\;I$. The desired contour for image segmentation is given by the zero level set of the steady state $\left(\frac{\partial \phi }{\partial t}=0\right)$.

### 2.2. Distance Regularized Level Set Evolution

It is well known that the finally found contour with the level set-based method is the zero level set of the level set function. However, in the traditional level set formulations, the level set function typically develops irregularities during its evolution, which may result in numerical errors and ultimately destroy the stability of the evolution [27]. In order to maintain the stability of the level set evolution, the traditional method is to reinitialize the level set function, which introduces other problems, such as how to apply this reinitialization. Therefore, Li et al. [27] proposed the distance regularized level set evolution (DRLSE), which makes use of a penalty term to make sure the level set function to be the signed distance function (at least in a vicinity of its zero level set) as far as possible.

The general energy formulation with the distance regularization is:

(6) $$\varepsilon \left(\phi \right)=\mu R_{p}\left(\phi \right)+\varepsilon_{ext}\left(\phi \right)$$

where ϕ is the level set function defined on a domain Ω, μ>0 is a constant, and $\varepsilon_{ext}\left(\phi \right)$ is the energy that depends on the image. $R_{p}\left(\phi \right)=\int_{\Omega }p\left(\left|\nabla \phi \right|\right)dx$ is the level set regularization term that enforces the level set evolution to be stable. To maintain the signed distance property |∇ϕ|=1, Li et al. [27] provide a double-well potential function:

(7) $$p\left(s\right)=\{\begin{array}{c} \frac{1}{{\left(2\pi \right)}^{2}}\left(1-\cos\left(2\pi s\right)\right),\;\mathrm{if}\;s\leq 1\\ \frac{1}{2}{\left(s-1\right)}^{2},\;\mathrm{if}\;s\geq 1\; \end{array}$$

To demonstrate the performance of DRLSE, Li et al. [27] applied it to the edge-based active contour model for the image segmentation. Finally, the energy functional is defined as follows:

(8) $$\varepsilon \left(\phi \right)=\mu R_{p}\left(\phi \right)+\lambda L_{g}\left(\phi \right)+\alpha A_{g}\left(\phi \right)$$

where λ>0 and α∈R are constant. $L_{g}\left(\phi \right)$ and $A_{g}\left(\phi \right)$ are defined by:

(9) $$L_{g}\left(\phi \right)≜\int_{\Omega }g\delta \left(\phi \right)\left|\nabla \phi \right|dx$$

and:

(10) $$A_{g}\left(\phi \right)≜\int_{\Omega }gH\left(-\phi \right)dx$$

The energy $L_{g}\left(\phi \right)$ computes the line integral of the function g along the zero level contour ϕ. The energy $A_{g}\left(\phi \right)$ is a weighted area of the region. Here, g is defined as Equation (5), δ and *H* are the Dirac delta function and the Heaviside function, respectively. The energy functional Equation (8) can be approximately minimized by solving the following equation:

(11) $$\begin{array}{c} \frac{\partial \phi }{\partial t}=\mu div\left(d_{p}\left(\left|\nabla \phi \right|\right)\nabla \phi \right)\;\\ \;+\lambda \delta_{\varepsilon }\left(\phi \right)div\left(g\frac{\nabla \phi }{\left|\nabla \phi \right|}\right)+\alpha g\delta_{\varepsilon }\left(\phi \right) \end{array}$$

with a given initial level set function ϕ(0,x,y). $\delta_{\varepsilon }\left(\cdot \right)$ is a approximate Dirac delta function. $d_{p}\left(\cdot \right)$ is a function defined as $d_{p}\left(s\right)=\frac{p^{\prime }\left(s\right)}{s}$. Equation (11) is considered as an edge-based geometric active contour model. For details about DRLSE readers may refer to [27].

### 2.3. Motivation

**Figure 1:**
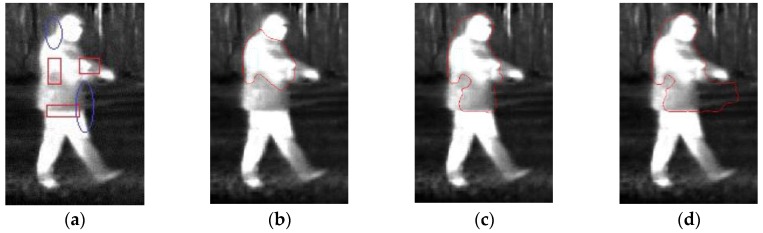
DRLSE segmentation results. (**a**) Original image; (**b**) α=2.5; (**c**) α=2.7; (**d**) α=3.

Infrared thermal images are generated by collecting the infrared radiation from the scenes. In some cases, the temperature difference of some part of the target and the background scene is not notable, which may cause weak target boundaries. Because the infrared energy emitted from different parts of a target may be significantly different, the intensity in the target region is inhomogeneous. The former may cause the boundary leakage problem. Meanwhile, the later may results in that the contour evolution prematurely stop, and then the boundary is formed in the interior of the target region. For example, there is an infrared image as shown in Figure 1a, which is to be segmented via DRLSE. The parameter values are set as those in [27]. The segmentation results with α=2.5, α=2.7 and α=3 are shown in Figure 1b–d, respectively.

As shown in Figure 1a, it is obvious that the pixel intensity of each region marked with the rectangular-shaped curve is inhomogeneous. Therefore, the segmentation result (shown in Figure 1b) indicates that the evolving contour stops at a wrong boundary that lies in the interior of the object. In order to reduce the intensity inhomogeneity effect, we increase the value of the parameter α. It can be seen from Figure 1c,d that the intensity inhomogeneity problem has been partially overcome by this approach. However, we find that the contour does not stop at the weak boundaries that are in the region circled with the oval-shaped curves as shown in Figure 1a.

To address the boundary leakage problem, Pratondo et al. [19] proposed a robust edge-stop function (ESF) for medical image segmentation, which is constructed by using the edge information from the image gradient values and some probability scores from a classifier. That is, the training samples are selected from the background and object regions of the image to be segmented, and then a classifier is trained to classify each pixel. The resulting probability scores are utilized to construct the ESF. However, for an infrared like the one shown in Figure 1a, the difference between the pixel intensities of some target regions and that of the background is not remarkable, so that it may be difficult to classify them into two categories. Therefore, in this paper, we propose the IALSE that measures the intensity change in the vicinity of the boundary and then reduces the intensity inhomogeneity in the target region to address the boundary leakage and intensity inhomogeneity problems, so that the edge-based contour evolution can be stopped at the desired boundary automatically.

## 3. Intensity Adjustment Level Set Evolution

### 3.1. Boundary Enhancement

Generally, the original image is smoothed by a Gaussian convolution kernel to reduce the noise and obtain an effective edge-stop function. However, in order to reduce the smooth effect on the edge, we use the following filter:

(12) $$G=\frac{1}{T}\cdot G_{\sigma ,N}\circ K_{N}$$

$G_{\sigma ,N}$ is a N×N filter generated by Gaussian function with the standard deviation σ. $K_{N}\;$is a N×N filter. For example, N=7, $K_{7}$ is defined as:

$$K_{7}=\left[\begin{array}{ccc} \begin{array}{ccc} 1 & 0 & 0\\ 0 & 1 & 0\\ 0 & 0 & 1 \end{array} & \begin{array}{c} 1\\ 1\\ 1 \end{array} & \begin{array}{ccc} 0 & 0 & 1\\ 0 & 1 & 0\\ 1 & 0 & 0 \end{array}\\ \begin{array}{ccc} 1 & 1 & 1 \end{array} & 1 & \begin{array}{ccc} 1 & 1 & 1 \end{array}\\ \begin{array}{ccc} 0 & 0 & 1\\ 0 & 1 & 0\\ 1 & 0 & 0 \end{array} & \begin{array}{c} 1\\ 1\\ 1 \end{array} & \begin{array}{ccc} 1 & 0 & 0\\ 0 & 1 & 0\\ 0 & 0 & 1 \end{array} \end{array}\right]$$

where “∘” is the Hadamard product. $\frac{1}{T}$ is a normalized factor such that the sum of all elements of the filter G equals to one.

**Figure 2:**
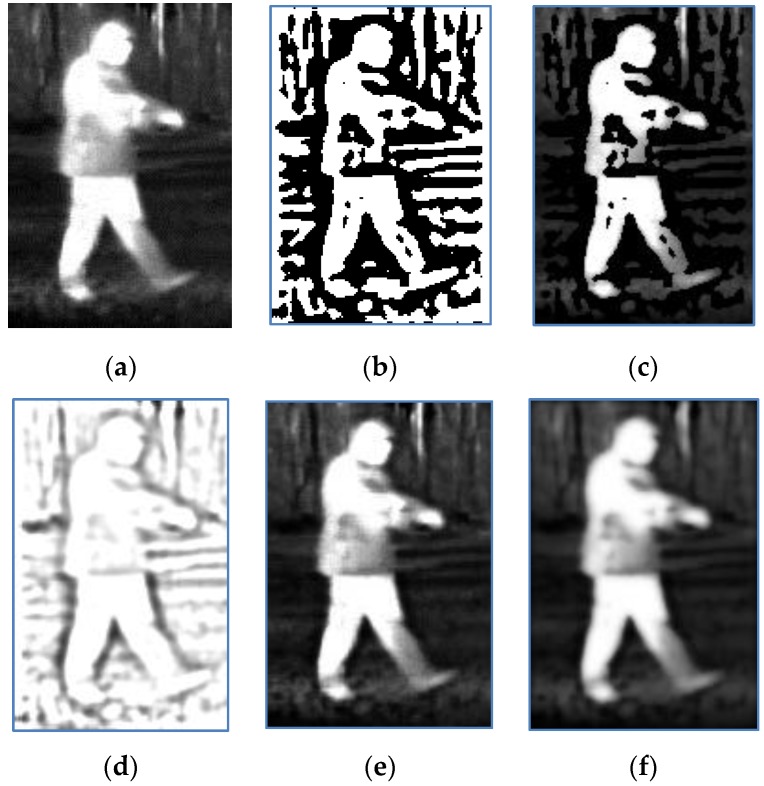
(**a**) Original image; (**b**) One-bit image; (**c**) Original image masked with (b); (**d**) Soft mark image; (**e**) Original image marked with (d); (**f**) Smoothed image marked with (d).

Natarajan [32] introduced a 17×17 convolution kernel $K_{o}\;$as follows:

$$K_{o}\left(i,j\right)=\{\begin{array}{cc} 1/25, & i,j\in \left\{0,\;4,\;8,\;12,\;16\right\}\\ 0, & otherwise \end{array}$$

from which they constructed the one-bit transform for the block-based motion estimation. Erturk [33] proposed a diamond-shaped structured filtering kernel $K_{r}$, which can be considered as the rotated version the filter $K_{o}$. Afterwards, Erturk [34] has applied this kernel based one-bit transform to the interesting region extraction in infrared images. The one-bit image is generated by comparing the original image I with the filtered image $I_{K_{r}}=I*K_{r}$ as follows:

(13) $$B\left(i,j\right)=\{\begin{array}{cc} 1, & I\left(i,j\right)\geq I_{K_{r}}\left(i,j\right)\\ 0, & \mathrm{otherwise} \end{array}$$

where (i,j) is the location of each pixel in an image. As shown in Figure 2. Figure 2b is the one-bit image, and Figure 2c is the masked infrared image. It can be seen that the one-bit transform can locate the target. However, some regions in the interior of the target are marked as black regions, which mean they do not belong to the target. The major reason is the intensity inhomogeneity in the vicinity of those regions.

Instead of utilizing the hard mark Equation (13), we introduce a soft mark *M* whose element is defined as:

(14) $$M\left(i,j\right)=\{\begin{array}{cc} 1, & if\;I_{G}\left(i,j\right)\geq I_{GK}\left(i,j\right)\\ {\left(\frac{I_{G}\left(i,j\right)}{I_{GK}\left(i,j\right)}\right)}^{q}, & otherwise \end{array}$$

$I_{G}$ is the smoothed image with the modified Gaussian filter in Equation (12). $I_{GK}\left(i,j\right)$ is obtained by filtering the image $I_{G}$ with the filter kernel *K* that is defined as follows:

$$K\left(i,j\right)=\{\begin{array}{cc} 1/25, & if\;i,j\in \left\{0,\;2,\;4,\;6,\;8\right\}\\ 0, & otherwise \end{array}$$

The filter *K* can be considered as the scaled version of the filter kernel $K_{o}$. The reason that we do not use the two filtering kernels $K_{o}$ and $K_{r}$ is that they have a larger spatial support, which means they will cover larger region in the infrared image. As we known in the edge detection, if we can find the edge by using both the filter with small size and the filter with large size, the former may be better than the latter for determining the edge position. Therefore, we introduce the filter *K* by down sampling (scale transform in the discrete version) the filter kernel $K_{o}$ by a factor 2 around the center. It should be noted that, if we replace $I_{G}$ and $I_{GK}$ with I and $I_{K_{r}}$ in Equation (14), respectively, when q→∞, the soft mark tends to be the hard mark Equation (13). When q=2, the soft mark (Equation (14)) for the image Figure 2a is shown in Figure 2d.

The infrared image and its smoothed image are marked with the soft mark as follows:

(15) $$\begin{array}{c} I_{M}=I\circ M\\ I_{GM}=I_{G}\circ M \end{array}$$

The original image Figure 2a and its smoothed image marked with the soft mark are shown in Figure 2e,f, respectively. By comparing Figure 2d with Figure 2b, and Figure 2e with Figure 2c, we find that the soft mark does not suddenly change the intensity as the hard mark, but smoothly weight the intensity in the vicinity of the edge. According to the Equation (15), if the intensity of M is lower (the region of pedestrian boundaries in Figure 2d), the corresponding intensity of $I_{M}$ will be lower too. As a result, compared with the original image, boundary of Figure 2e is clearer. It is obvious that a larger q will lead to a clearer boundary. However, the boundary is imperfect, and the intensity inhomogeneity becomes worse in the interior of the target region. In contrast, a smaller q may result in an unacceptable boundary enhancement, but prevent the intensity inhomogeneity from being worse, which is caused by weighting the intensity. Therefore, a soft mark with an appropriate parameter q will ensure that weighted intensity in the vicinity of the desired boundary is useful to stop the evaluating contour, and it is possible to adjust the intensity in the interior of the target region to address the intensity inhomogeneity problems. The parameter q is empirically set to be 2 in our experiments.

### 3.2. Intensity Adjustment

If there is intensity inhomogeneity in the original infrared image, the inhomogeneous intensity may lead to a pseudo boundary in the interior of the target region. Furthermore, the soft mark may aggravate the intensity inhomogeneity, because the soft mark values are less than one near the edges in the target region. Therefore, we will adjust the intensity to reduce the intensity inhomogeneity in this section. Intuitively, the intensity adjustment should dependent on the current intensity value and the image property. We adjust the marked image $I_{GM}$ as follows:

(16) $$I_{GMA}=I_{GM}\circ \left(1+f\left(I_{GM}\right)\right)$$

where $f\left(I_{GM}\right)$ is a weight function that controls the adjustment value. Its size is the same as that of$\;I_{GM}$.

Our approach relies on the assumption that the target region has a hotter (brighter) appearance than most of the background region. We let the maximum value and the average value of the marked image $I_{GM}$ to be $I_{max}$ and$\;I_{mean}$, respectively. It can be observed from the thermal infrared image that the intensity values of the darker target region are less than those of the brighter target region, but the majority of them are larger than most intense background values. Therefore, we can reasonably assume that the intensity values of the darker target region are close to or larger than the mean value $\;I_{mean}$, and assign larger weights to those pixels. Meanwhile, if the intensity value $I_{GM}\left(i,j\right)$ is closer to $I_{max}$, a smaller weight value should be assigned. In this paper, we make use of the normalized Gaussian function (Equation (17)) for the pixels whose intensity values belong to $\left[\;I_{mean},I_{max}\right]$, in which $x_{i,j}=\frac{I_{GM}\left(i,j\right)}{I_{max}}$ and $\mu =\frac{I_{mean}}{I_{max}}$. The variance of the normalized Gaussian function is set to be 0.2, which guarantees the adjusted intensity values are not larger than the maximum intensity value $I_{max}$.

Naturally, if the intensity value $I_{GM}\left(i,j\right)$ of a pixel is close to zero, it is overwhelmingly probable that the pixel belongs to the background. The weight ${\left[f\left(I_{GM}\right)\right]}_{i,j}$ should approach to zero. In order to maintain smooth change of the adjusted intensity values, we also utilize the normalized Gaussian function for the pixels whose intensity values belong to $\left[\;0,I_{mean}\right]$. Thus, the weight function $f\left(I_{GM}\right)$ is defined as:

(17) $${\left[f\left(I_{GM}\right)\right]}_{i,j}\;=\{\begin{array}{c} \frac{\left(\exp\left(-{\left(x_{i,j}-\mu \right)}^{2}/0.2\right)-\text{exp}\left(\frac{-{\left(1-\mu \right)}^{2}}{0.2}\right)\right)}{1-\text{exp}\left(\frac{-{\left(1-\mu \right)}^{2}}{0.2}\right)},1\geq x_{i,j}\geq \mu \\ \frac{\left(\exp\left(-{\left(x_{i,j}-\mu \right)}^{2}/0.2\right)-\text{exp}\left(\frac{-{\left(\mu \right)}^{2}}{0.2}\right)\right)}{1-\text{exp}\left(\frac{-{\left(\mu \right)}^{2}}{0.2}\right)},0\leq x_{i,j}<\mu \end{array}$$

**Figure 3:**
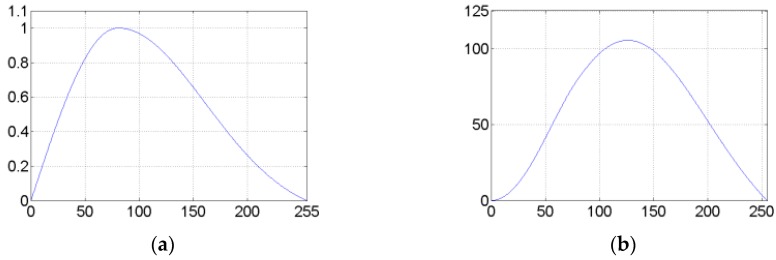
(**a**) Weight functions for the intensity value 0 to 255; (**b**) Adjustment value for the intensity value 0 to 255.

**Figure 4:**
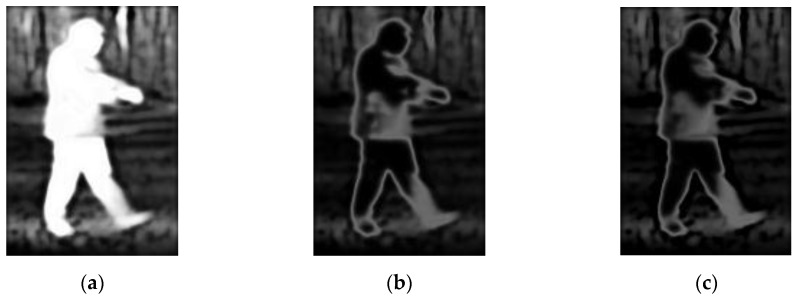
(**a**) Intensity adjusted image$\;I_{GMA}$; (**b**) The difference image between $I_{GMA}$ and$\;I_{GM}$; (**c**) The difference image between $I_{GMA}$ and $I_{G}$.

For example, the maximum value and the average value of the image shown in Figure 2f are $I_{max}=255$ and$\;I_{mean}=80.6$, respectively. The weight function and the adjustment value for the intensity value 0 to 255 are shown in Figure 3. The intensity adjusted image in Figure 4a. When comparing Figure 4a with the original image in Figure 2a, it is observed that the intensities of the target region become approximately uniform or change smoothly. The difference images in Figure 4b,c demonstrate the intensity change of each pixel, from which we find the wanted results that the intensities of the gray region of the target have been amplified. Meanwhile, it is shown in Figure 4a that the blurry boundary between the target and the background becomes more distinct. We should notice that the intensities of the background have also been enlarged. However, the background is much darker than the target, and we have enhanced the boundary. Therefore the target detection is still prominent.

After we obtain the processed image $I_{GMA}$, the edge indicator function is computed as:

(18) $$g\left(I_{GMA}\right)=\frac{1}{1+{\left|\nabla I_{GMA}\right|}^{2}}$$

By substituting Equation (15) into Equation (16), we have $I_{GMA}=I_{G}\circ M\circ \left(1+f\left(I_{G}\circ M\right)\right)$, if we let $P\left(I_{G}\right)=M\circ \left(1+f\left(I_{G}\circ M\right)\right)$, then there is:

(19) $$\nabla I_{GMA}=\nabla I_{G}\circ \left(P\left(I_{G}\right)\right)+I_{G}\circ \nabla \left(P\left(I_{G}\right)\right)$$

This means that, according to the image property, we adaptively adjust $\nabla I_{G}$ and obtain $\nabla I_{GMA}$. Then the resulting edge indicator function $g\left(I_{GMA}\right)$ will stop the evolving curve at the desired boundary.

### 3.3. Level Set Based Image Segmentation

The proposed infrared image segmentation algorithm consists of four steps: image smoothing; boundary enhancement; intensity adjustment, and level set based image segmentation. The segmentation algorithm is summarized as follows:

Input: the infrared image I.

Segmentation:

1. Image Smoothing. Using the filter G (Equation (12)), smooth the image I and obtain the image$\;I_{G}$.
2. Boundary Enhancement. Compute the soft mark M(i,j) (Equation (14)) for the image $I_{G}$. Then the boundary enhanced image $I_{GM}$ is obtained with Equation (15).
3. Intensity Adjustment. Calculate the weight $f\left(I_{GM}\left(i,j\right)\right)$ for each pixel with Equation (17). Adjust the intensity of the image $I_{GMA}$ with Equation (16).
4. Level Set Based Image Segmentation. Generate the edge stop function $g\left(I_{GMA}\right)$ with Equation (18). Equation (11) is applied to carry on level set evolution and get the infrared image segmentation result, in which the terminal condition is that the evolving contour is not change for five iterations, or the number of the iterations reaches to the set value.

Output: The segmentation result.

## 4. Experimental Results and Discussions

In this section, we present the experimental results of the proposed method on the benchmark infrared images. Meanwhile, in order to demonstrate the effectiveness of the proposed algorithm, we also compare it with the state-of-the-art approaches.

### 4.1. Data Set and Evaluation Measures

In order to fully test the performance of the proposed method, we chose thermal infrared images from different benchmark datasets, that is, OSU Thermal Pedestrian Database (OSUT) [35], Terravic Motion IR Database (TMID) [36], Pedestrian Infrared/visible Stereo Video Dataset (PISVD) [37] and Infrared Action Recognition [38], because the images of different datasets are captured with different devices under different environments. Pedestrian regions in OSUT are small and the images are filled with noise. When TMID pedestrians are indoors, filament lamps seriously affect the segmentation. At the same time, the intensity of some regions of pedestrians is similar to the background. When pedestrians in TMID are outdoors, the intensities of both background and pedestrians are inhomogeneous. However, the pedestrian intensity is also homogeneous in some images. Pedestrians in PISVD are indoors and the intensity of pedestrians is severely inhomogeneous. There is little noise in the Infrared Action Recognition images, but the floors reflect pedestrians’ reflections and the intensity of pedestrians is also inhomogeneous. The sizes of images in these datasets are 360 × 240, 320 × 240, 480 × 360 and 293 × 256 pixels, respectively. The ground truths of these images are drawn by an expert.

The accuracy and precision of image segmentation evaluation can be broadly classified into distance-based coefficients, region-based coefficients, and statistical analyses of the entire images [39]. In this paper, we adopt five measures for characterizing the performance of the proposed infrared image segmentation algorithm. They are the Dice coefficient, also well known as the similarity index (SI), Jaccard index (JI), Hausdorff Distance, Conformity Coefficient and Area Overlapped Error (AOE) [40].

Let $\Omega_{1}$ and $\Omega_{2}$ stand for two intersection sets (if $\Omega_{1}$ and$\;{\;\Omega }_{2}$ do not intersect, we also consider they intersect, but the area of the intersection is zero). The Jaccard coefficient (JI) measures the ratio of the intersection area of $\Omega_{1}$ and${\;\Omega }_{2}$ divided by the area of their union:

(20) $$\mathrm{JI}=\frac{\left|\Omega_{1}\cap^{\text{​}}\Omega_{2}\right|}{\left|\Omega_{1}\cup^{\text{​}}\Omega_{2}\right|}$$

The Dice coefficient (SI) is calculated as the ratio of the intersection area divided by the sum of each individual area:

(21) $$\mathrm{SI}=\frac{2\left|\Omega_{1}\cap^{\text{​}}\Omega_{2}\right|}{\left|\Omega_{1}\right|+\left|\Omega_{2}\right|}$$

The Hausdorff Distance is a kind of distance-based coefficient, which is defined as:

(22) $$H\left(\Omega_{1},\Omega_{2}\right)=\max\left\{h\left(\Omega_{1},\Omega_{2}\right),h\left(\Omega_{2},\Omega_{1}\right)\right\}$$

where $h\left(\Omega_{1},\Omega_{2}\right)=sup_{a\in \Omega_{1}}inf_{b\in \Omega_{2}}||a-b||$ and ||·|| is the chosen norm. It measures the distance between the segmentation contour (or surface in 3D) and the true boundary, and is used when the delineation of the boundary is critical.

Conformity Coefficient is a coefficient that measures the global similarity and can be expressed in terms of JI or SI as follows:

(23) $$K_{c}=\frac{2\;\mathrm{JI}-1}{\mathrm{JI}}=\frac{\left(3\;\mathrm{SI}-2\right)}{\mathrm{SI}}$$

Area Overlapped Error (AOE) measures between $\Omega_{1}$ and $\Omega_{2}$ as follows:

(24) $$\mathrm{AOE}=\left(1-\left(\frac{\Omega_{1}\cap^{\text{​}}\Omega_{2}}{\left(\Omega_{1}+\Omega_{2}\right)-\left(\Omega_{1}\cap^{\text{​}}\Omega_{2}\right)}\right)\right)$$

It should be noted that both SI and JI have a minimum score of zero when there is no intersection at all, but the conformity coefficient $K_{c}$ is always smaller than the other two coefficients and has a much wider range of index scores (−∞,1], so we also choose the Conformity coefficient to evaluate the accuracy and precision of the proposed algorithm. According to the Equations (20)–(24), we can find that the larger the JI, SI and${\;K}_{c}$, (the upper bound is 1) or the less H(A,B) and AOE, the better the segmentation result achieved.

### 4.2. Experimental Setting

The infrared image segmentation algorithms have been implemented in the MATLAB 2017a environment on a computer with an Intel Xeon E5-2687W v2 3.4 GHz CPU ×2 CPU and 64 GB RAM. In the experiments of our proposed method, we set the smoothing filter parameters N=7 and σ=2, the soft mark parameter q=2. The parameters of the level set method are set as those in [27], except for the parameter α which is adjusted according to the segmented infrared images.

### 4.3. Comparisons of Edge Stop Function

**Figure 5:**
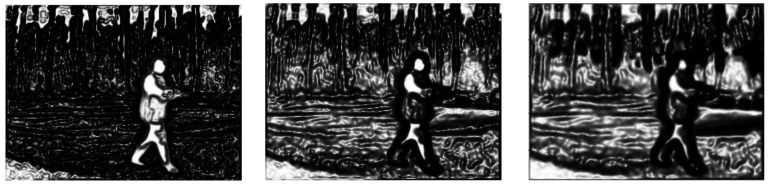

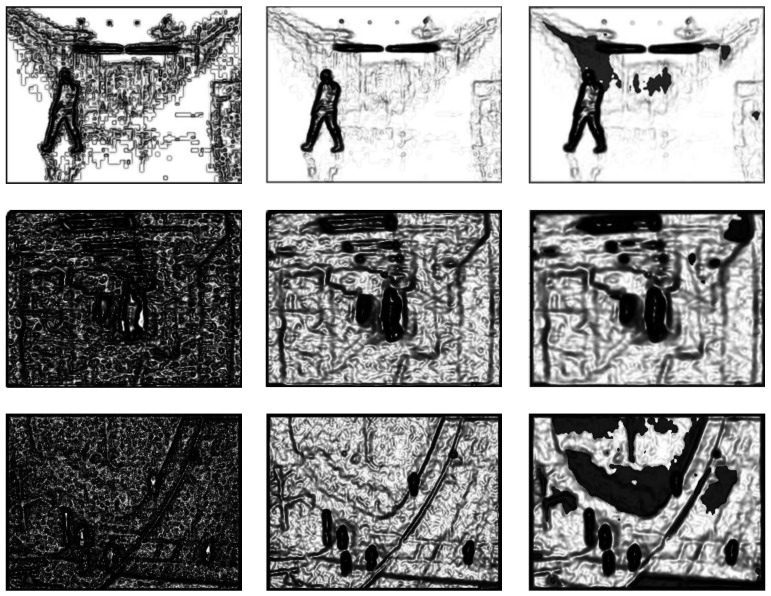
Edge indicator functions are generated with the proposed method (**the first column**); DRLSE (**the second column**); Robust_ESF (**the third column**).

As summarized in Section 3, the purpose of IALSE is to generate a more robust edge indicator function so that the evolving curve can stop at the desired boundary. We convert the edge stop functions generated by IALSE, DRLSE and Robust_ESF into grey images that are shown in Figure 5, in which the value of the indicator function is proportional to the intensity value.

As stated in [5] and the energy minimization principle in Equation (11), when the desired boundary has high variations on the edge indicator function, the contour will stop at those positions. It can be seen from Figure 5 that the edge indicator function on the target regions has larger and uniform values, and has high variations around the boundary. Therefore, the edge indicator function of this paper is better than those of the two compared methods.

### 4.4. Method Comparison

**Figure 6:**
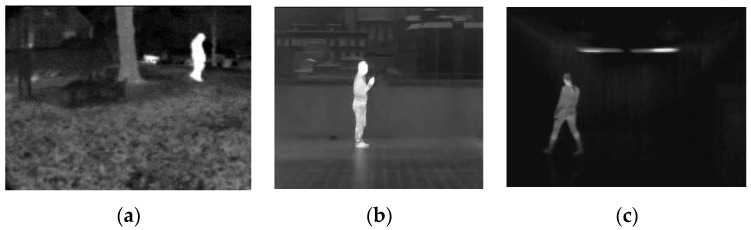

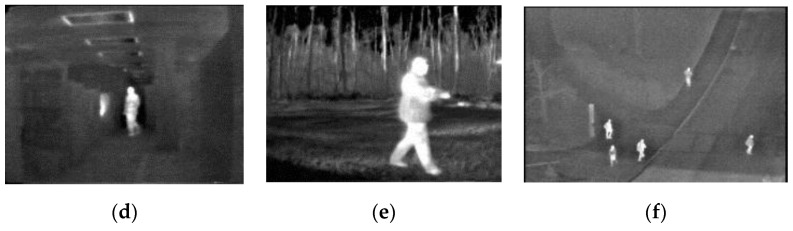
Source thermal infrared images.

In recent years, many kinds of level set or active contour algorithms have been proposed for image segmentation. To demonstrate the effectiveness of the proposed algorithm, we compared our method (IALSE) with other segmentation methods, that is, DRLSE from Li et al. [27], Robust_ESF in Pratondo et al. [19], FCMLSM in Li et al. [41], and LSACM from Zhang et al. [42]. In Figure 6, the intensity in the target region of the first image seems approximately uniform from the human visual cognition, which can be well segmented with some existed level set based methods. 

**Figure 7:**
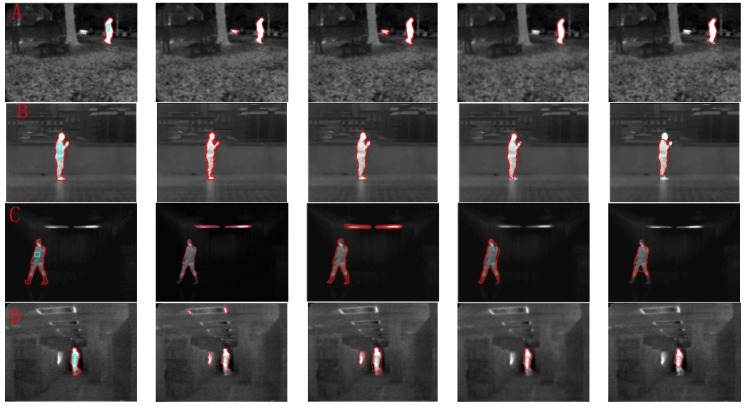

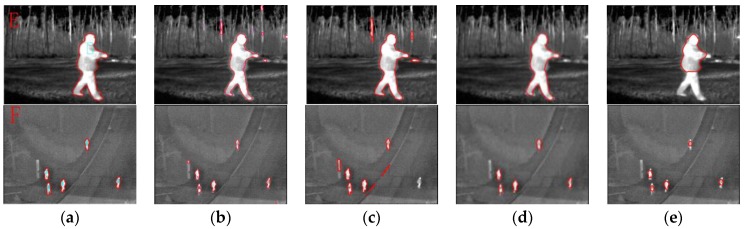
Segmentation results. From left to right (**a**) The proposed method IALSE (rectangle stands for the initial curve); (**b**) FCMLSM; (**c**) LSACM; (**d**) Robust_ESF; (**e**) DRLSE.

We make use of them to testify whether our processes can improve the segmentation on the infrared images with high quality targets. For the other five images, it is obvious that the intensity is inhomogeneous in the target region, from which we demonstrate that our method can obtain satisfied segmentation results. The segmentation results of proposed and compared methods are shown in Figure 7. It can be seen from the first row of Figure 7 that the targets can be well segmented with IALSE, FCMLSM and Robust_ESF. However, the FCMLSM and LSACM methods have segmented some background regions into the target regions. Therefore, our proposed method can work well on the infrared images with high quality targets. It is found from the second row that there is intensity inhomogeneity in the target region, but the target region is distinct from the background. Thus the target is well segmented by using the first four methods, in which FCMLSM is the best. For the infrared image with intensity inhomogeneity (third to sixth rows), our method is better than the other approaches. As shown in the third row of Figure 7, the proposed method extracts the target very well. However, due to the intensity inhomogeneity, the segmentation results with other methods exclude the foot and legs regions that are covered by the boots. In the sixth row of Figure 7, five target regions have been well segmented with our introduce method. The target at the down left corner has been segmented with FCMLSM into two regions that are separated by a region with lower intensity value. Meanwhile the method LSACM has lost a target region. The Robust_ESF method is designed to solve the poorly defined boundary problem. Therefore our method is better than other methods on the infrared images with intensity inhomogeneity.

### 4.5. Quantitative Evaluation

**Figure 8:**
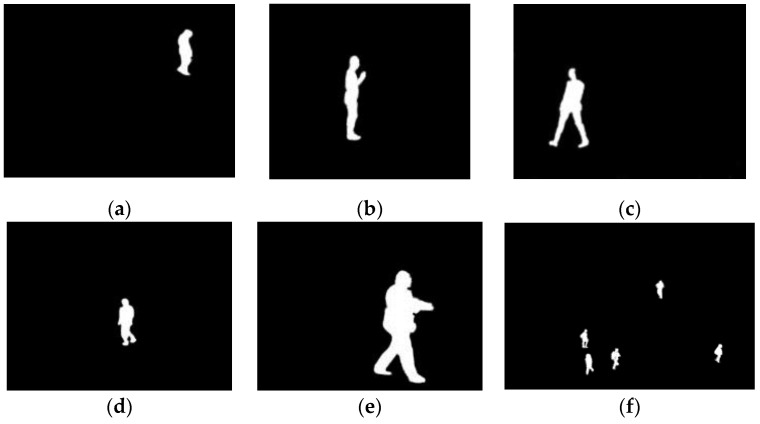
Ground truths.

**Figure 9:**
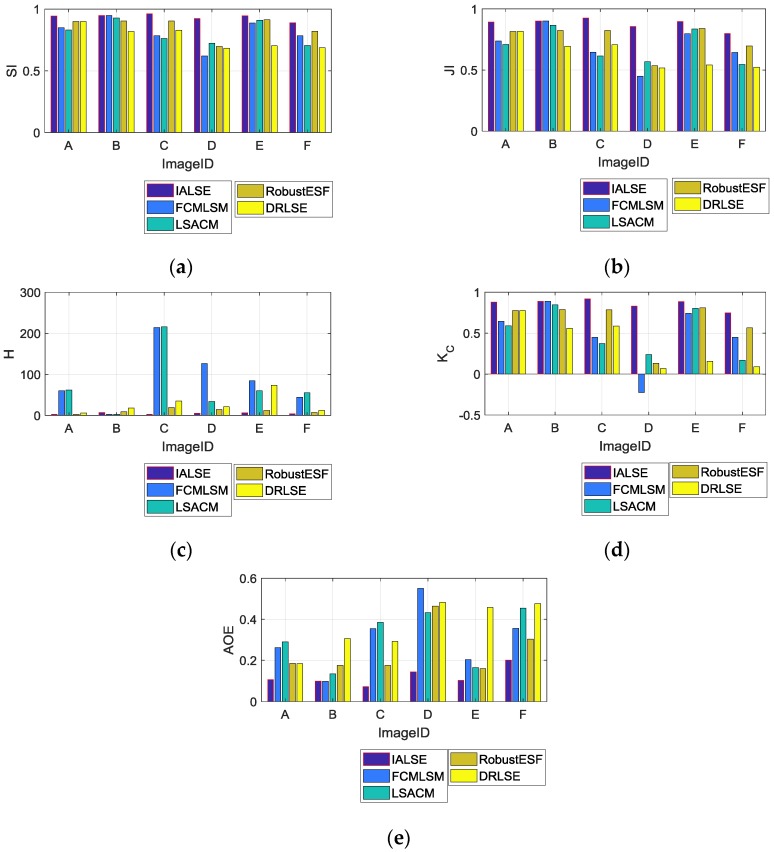
Bar graph of quantitatively analyzed segmentation result of infrared images. (**a**) SI, (**b**) JI, (**c**) H, (**d**)K_c, (**e**) AOE.

In order to objectively evaluating the performance of the introduced method, we make use of five measurements JI, SI, H,${\;K}_{c}$ and AOE. All the measurements are calculated with the segmentation result and the ground truth. The ground truths of images (in Figure 6) are drawn by an expert, which are shown in Figure 8. The calculated measurement values for IALSE and compared methods are listed in Table 1, in which the best measurement value for each image has been marked in bold. It should be noted that if some regions in the background are also segmented into the target regions, the measurement values for corresponding methods are worse than those of the proposed method. To make the evaluation data more intuitive, we express the above evaluation data of Table 1 in the form of bar graph in Figure 9.

Due to the boundary enhancement and intensity adjustment, the thermal infrared images are well segmented with our proposed method. At the same time, the side product is that the curve evolution of our method will automatically stop at the desired boundary for all experimental images. The number of iterations that the proposed method achieves the segmentation results is listed in Table 2.

**Figure 10:**
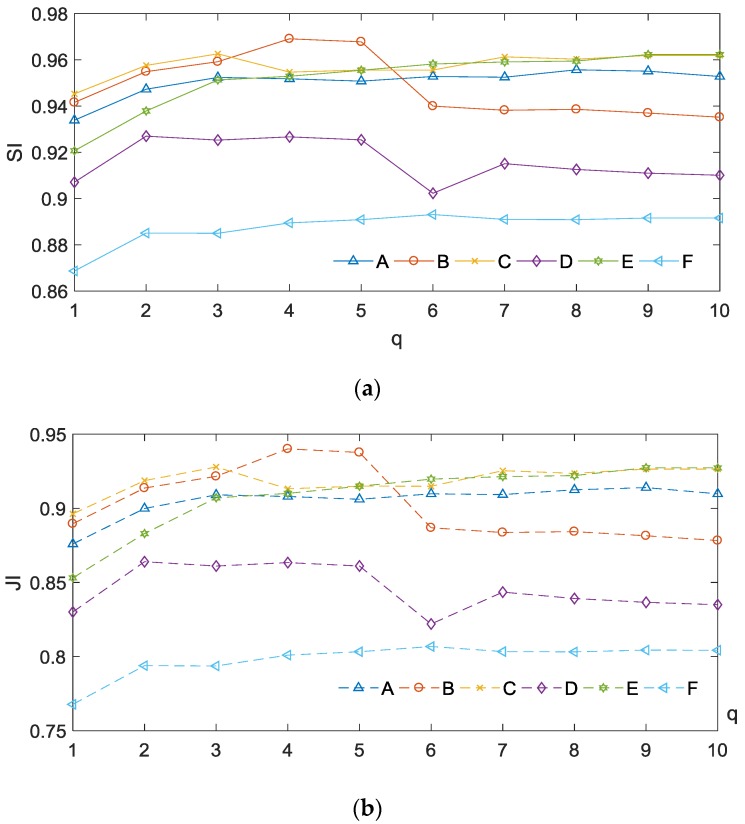
Segmentation accuracy index with different parameter values *q* (**a**) SI and (**b**) JI.

**Figure 11:**
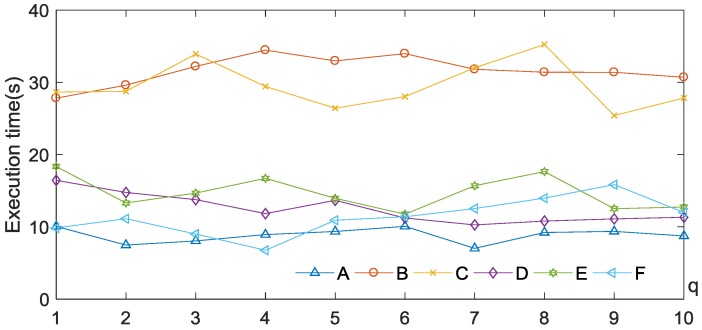
Average execution time of proposed method with different q in different images.

We conducted the experiments on infrared images (in Figure 6) with different parameter values q=1, 2,⋯, 10, in which the parameter α is adjusted from 0.5 to 10 with step size 0.2. The best segmentation accuracy of each q, evaluated by SI and JI, are given in Figure 10. It can be seen that it is difficult to find an optimal parameter q for all experimental images. However, the segmentation results with q=2 are better than those with q=1. At the same time, we also offer the average CPU times with different q of proposed method in different experimental images which is shown in Figure 11. In order to make the data more reasonable, we repeat the experiment 10 times and take the average. We can find that, compared with other value, the speed of proposed method q=2 is not the slowest or even faster than most of the experiments with other values of *q*. Therefore we set q=2 to compared with other existing methods. One of our aims is to reduce the intensity inhomogeneity.

**Figure 12:**
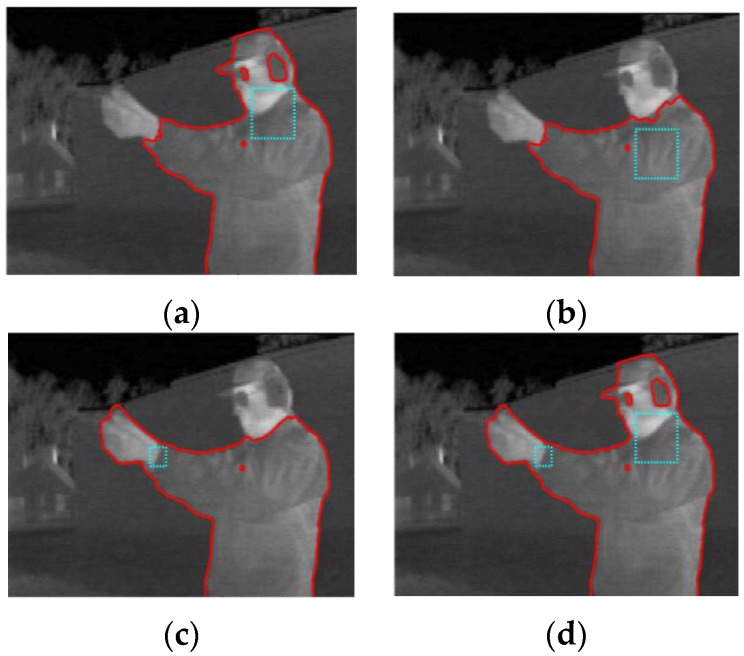
Segmentation results with different initial contours (cyan lines stand for the initial curves). (**a**) Segmentation result with initial contour around the neck. (**b**) Segmentation result with initial contour around the shoulder. (**c**) Segmentation result with initial contour around the wrist. (**d**) Segmentation result with initial contour around the shoulder and wrist.

However, our method is still sensitive to initialization for the image with significant intensity inhomogeneity in the target region. As shown in Figure 12, we have obtained different segmentation results under the different initial curves while other parameters are the same. We can see that the body covered by cloth can be segmented well even though the intensity is similar to the background. Besides, if we want to segment the hands or head, the region of initial contour have to include part of the regions of hands or head. Because the intensities of hands and head are different from those of the body, an appropriate initialization is necessary to obtain the satisfied segmentation result.

## 5. Conclusions

In this work, we have introduced a robust thermal infrared image segmentation method based on the level set formulation, which we call IALSE. The proposed IALSE method can increase the contrast between target regions and the background, and adjust the intensity of the target region to be more homogeneous in the infrared image. These strategies can make the curve evolution stop at the desired boundary automatically. To evaluate the proposed method for the infrared image segmentation, we conducted experiments on the thermal infrared images chosen from three benchmark datasets, and compared our method with some existing methods. By using the subjective evaluation and the objective measurements, the superior performance of our method has been demonstrated.

## Figures and Tables

**Table 1:** Calculation data of comparison methods and proposed method.

Image		A	B	C	D	E	F	Average
IALSE (proposed)	SI	**0.9431**	0.9476	**0.9617**	**0.9229**	**0.9457**	**0.8885**	**0.9349**
	JI	**0.8923**	0.9005	**0.9263**	**0.8568**	**0.8970**	**0.7993**	**0.8787**
	H	**2.8242**	7.0711	**3.1623**	**5.6569**	**6.7082**	**4.1231**	**4.9243**
	$K_{c}$	**0.8794**	0.8895	**0.9204**	**0.8328**	**0.8852**	**0.7490**	**0.8594**
	AOE	**0.1077**	0.0995	**0.0737**	**0.1432**	**0.1030**	**0.2007**	**0.1213**
FCMLSM	SI	0.8490	**0.9480**	0.7842	0.6201	0.8866	0.7839	0.8120
	JI	0.7376	**0.9012**	0.6451	0.4494	0.7962	0.6447	0.6957
	H	60.008	**3**	214.3	126.7	84.314	44.294	88.769
	$K_{c}$	0.6442	**0.8903**	0.4498	−0.2252	0.7441	0.4488	0.492
	AOE	0.2624	**0.0988**	0.3549	0.5506	0.2038	0.3553	0.3043
LSACM	SI	0.8301	0.9284	0.7618	0.7241	0.9106	0.7061	0.8102
	JI	0.7096	0.8663	0.6153	0.5676	0.8358	0.5458	0.6901
	H	62.097	3	215.78	34	60.1082	55.3624	71.725
	$K_{c}$	0.5907	0.8457	0.3747	0.2380	0.8036	0.1677	0.5034
	AOE	0.2904	0.1337	0.3847	0.4324	0.1642	0.4542	0.3099
Robust_ESF	SI	0.8989	0.9039	0.9036	0.6973	0.9134	0.8215	0.8564
	JI	0.8164	0.8247	0.8242	0.5353	0.8407	0.6971	0.7564
	H	3.1623	9.0554	19.417	14.422	12.083	7.2801	10.903
	$K_{c}$	0.7751	0.7874	0.7867	0.1319	0.8105	0.5655	0.6429
	AOE	0.1854	0.1753	0.1758	0.4647	0.1593	0.3029	0.2439
DRLSE	SI	0.8991	0.8195	0.8288	0.6822	0.7033	0.6869	0.7637
	JI	0.8166	0.6942	0.7076	0.5176	0.5424	0.5232	0.6265
	H	5.8310	18.028	35.128	21.024	73.682	12	27.479
	$K_{c}$	0.7755	0.5595	0.5868	0.0682	0.1563	0.0885	0.3443
	AOE	0.1834	0.3058	0.2924	0.4824	0.4576	0.4768	0.3664

**Table 2:** Number of iterations of the proposed method.

Image	A	B	C	D	E	F
Number of Iterations	415	800	630	560	1035	350
